# A Platform-Independent Method for Detecting Errors in Metagenomic Sequencing Data: DRISEE

**DOI:** 10.1371/journal.pcbi.1002541

**Published:** 2012-06-07

**Authors:** Kevin P. Keegan, William L. Trimble, Jared Wilkening, Andreas Wilke, Travis Harrison, Mark D'Souza, Folker Meyer

**Affiliations:** 1 Argonne National Laboratory, Argonne, Illinois, United States of America; 2 University of Chicago, Chicago, Illinois, United States of America; 3 Institute for Genomics and Systems Biology, Chicago, Illinois, United States of America; Accelrys, United States of America

## Abstract

We provide a novel method, DRISEE (duplicate read inferred sequencing error estimation), to assess sequencing quality (alternatively referred to as “noise” or “error”) within and/or between sequencing samples. DRISEE provides positional error estimates that can be used to inform read trimming within a sample. It also provides global (whole sample) error estimates that can be used to identify samples with high or varying levels of sequencing error that may confound downstream analyses, particularly in the case of studies that utilize data from multiple sequencing samples. For shotgun metagenomic data, we believe that DRISEE provides estimates of sequencing error that are more accurate and less constrained by technical limitations than existing methods that rely on reference genomes or the use of scores (e.g. Phred). Here, DRISEE is applied to (non amplicon) data sets from both the 454 and Illumina platforms. The DRISEE error estimate is obtained by analyzing sets of artifactual duplicate reads (ADRs), a known by-product of both sequencing platforms. We present DRISEE as an open-source, platform-independent method to assess sequencing error in shotgun metagenomic data, and utilize it to discover previously uncharacterized error in *de novo* sequence data from the 454 and Illumina sequencing platforms.

## Author Summary

Sequence quality (referred to alternatively as the level of sequencing error or noise) is a primary concern to all sequence-dependent investigations. This is particularly true in the field of metagenomics where automated tools (e.g. annotation pipelines like MG-RAST) rely on high fidelity sequence data to derive meaningful biological inferences, and is exacerbated by the capacity of next generation sequencing platforms that continue to expand at a rate greater than Moore's law. We demonstrate that the most commonly utilized means to assess sequencing error exhibit severe limitations with respect to analysis of metagenomic data. Furthermore, we introduce a method (DRISEE) that accounts for these limitations through the application of a novel approach to assess sequencing error. DRISEE-based analyses reveal previously unobserved levels of sequencing error. DRISEE provides a platform independent measure of sequencing error that objectively assesses the quality of entire sequence samples. This assessment can be used to exclude low quality samples from computationally expensive analyses (e.g. annotation). It can also be used to evaluate the relative fidelity of analyses after they have been performed (e.g. annotation of error prone samples is less reliable than that of samples with low levels of sequencing error).

## Introduction

Accurate quantification of sequencing error is the single most essential consideration of sequence-dependent biological investigations. While true of all investigations that utilize sequencing data, this is particularly true with respect to metagenomics. Metagenomic studies produce biological inferences as the near-exclusive product of computational analyses of high throughput sequence data that attempt to classify the taxonomic (through 16s ribosomal amplicon sequencing [MG-RAST [1], QIIME [2]]) and functional (through whole genome shotgun sequencing [MG-RAST [1]]) content of entire microbial communities. The accuracy of these inferences rests largely on the fidelity of sequence data, and consequently, on the ability of existing methods to quantify and account for sequencing error. Surprisingly, the most widespread methods to determine sequencing-error in metagenomic data lack essential features and/or produce underestimates of the overall error that disregard a substantial portion of sequencing-related experimental procedures.

Sequence-based experimental inferences, particularly those related to the identification and characterization of features (protein or 16s rRNA coding regions, regulatory elements, etc.) are greatly affected by the presence of sequencing errors [3]. Errors in metagenomic amplicon-based sequencing have led to grossly inflated estimates of taxonomic diversity [4], [5], [6]. While methods such as denoising [2], [7], [8] have been developed to address these issues in *amplicon-based* metagenomic sequencing [2], [9], no analogous techniques have been reported to account for noise/error in the context of *shotgun-based* metagenomic sequencing. Limitations inherent to methods used to assess *de novo* sequencing error are largely to blame. At present, two methods are commonly used: reference-genome and score -based.

Reference-genome-based methods compare *de novo* sequenced reads to preexisting standards (published genomes). Samples are typically cultured from a clonal isolate for which a reliable reference genome is readily available. Sequenced reads undergo an initial alignment to the selected reference genome to match *de novo* sequences with the regions in the reference genome to which they correspond. Reads that do not exhibit a high enough level of identity with the reference genome are excluded from further analysis. Reads that exhibit a high fidelity match to a region in the reference genome are compared to that region in great detail. Deviations between sequenced reads and their corresponding loci in the reference genome are scored as errors; these are typically reported with respect to frequency and type (i.e. insertion, deletion, substitution). Selection of the most appropriate reference genome is essential. This is problematic when the best available reference is a related strain or species. In these cases, real biological variation can be mistaken for sequencing error [10], [11], [12], [13]. Reference-genome-based methods provide a particularly effective means to examine sequencing error in the context of genomic (i.e. single genome sequencing or re-sequencing) data, but are not applicable to metagenomic samples as these typically contain enormous taxonomic diversity (samples contain a broad spectrum of species) for which little adequate reference data is available. Many species have no appropriate reference genome(s), and reference metagenomes do not currently exist.

**Figure 1:**
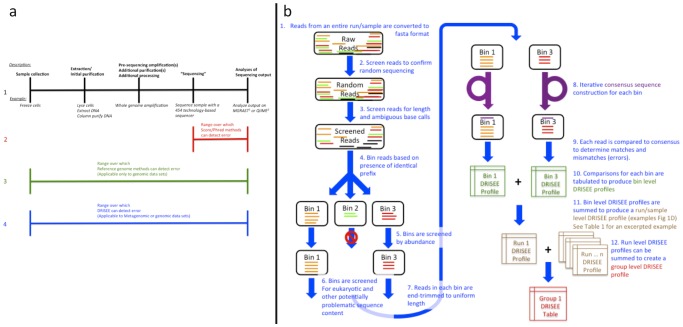
(a) Error detection capabilities of Score, Reference-genome, and DRISEE methods. (1) Simplified procedural diagram of a typical sequencing protocol. **Sample collection**: First, the biological sample is collected, **Extraction/Initial purification**: Then the RNA/DNA undergoes extraction and initial purification procedures, **Pre-sequencing amplification(s)**: Next, the extracted genetic material may undergo amplification (e.g. whole genome amplification – see main text) followed by additional purifications and/or other processing procedures, **“Sequencing”**: Genetic material is placed in the sequencer itself, and is sequenced. Note that sequencing itself frequently involves additional rounds of amplification, **Analyses of sequencing output**: Sequencer outputs are analyzed. (2) Given a procedure such as A, the portion of the procedure over which score/Phred-based methods can detect error is indicated in red. (3) Given a procedure such as A, the portion of the procedure over which reference-genome-based methods can detect error is indicated in green. Note that reference-genome-based methods are only applicable to single genome data; they cannot consider metagenomic data. (4) Given a procedure such as A, the portion of the procedure over which DRISEE-based methods can detect error is indicated in blue. Note that DRISEE methods can be applied to metagenomic or genomic data, provided that certain requirements are met. See methods. 1: BMC Bioinformatics. 2008 Sep 19;9:386. 2: Nat Methods. 2010 May;7(5):335–6. Epub 2010 Apr 11. **(b) DRISEE workflow** The steps in a typical DRISEE workflow are depicted and briefly described (in figure captions). Please see Text S1 (Supplemental Methods, *Typical DRISEE workflow*) for a much more detailed description of each depicted step.

Score-based methods use an alternative approach. Sequencer signals are compared with sophisticated, frequently proprietary, probabilistic models that attempt to account for platform-dependent artifacts, generating base calls, each with an affiliated quality (Phred or Q score) that provides an estimate of error frequency, but no information regarding error type. Although score-based methods are applicable to metagenomic data, their inability to consider error type can prove to be a substantial limitation. For example, similarity-based gene annotation is extremely sensitive to frame-shifting insertion/deletion errors but only moderately affected by substitutions [3]. In this context, knowledge of error type, specifically the ratio of insertion and/or deletions to substitutions provides crucial information, knowledge unattainable with conventional Phred or Q scores. The absence of information regarding error type is an even greater concern in light of documented platform-dependent biases in sequencing error type: Illumina-based sequencing exhibits high substitution rates [14], whereas 454 technologies exhibit a preponderance of insertion/deletion errors [13]; identical Q scores from these two technologies are likely to represent different types of error, rendering ostensibly similar metrics incomparable [13], [15], [16], [17], [18], [19], [20]. The most concerning, but paradoxically least discussed and perhaps least understood, deficit of score-based methods is their implicit disregard of experimental procedure. Typical sequencing efforts employ a host of procedures to extract, amplify, and purify genetic material, experimental processes that necessarily contribute errors (i.e. introduction of non-biological bias in sequence content and/or abundance relative to original biological template sequences); however, as these errors are introduced before the actual act of sequencing, they can not be accounted for with score-based methods. Thus, a large portion of experimental error in sequencing is frequently overlooked (Figure 1a) (an in depth literature search revealed no works that directly address this issue).

Reference-genome and score-based sequencing error determination methods require extensive prior knowledge in the form of reference genomes and/or elaborate platform dependent error models. At present it is not possible to apply reference-genome-based methods to metagenomic data. Score-based methods provide, at best, an incomplete assessment of error that is incomparable between technologies and provides no information with respect to error type. Neither of these approaches is well suited to platform-independent analysis of error in shotgun-based metagenomic data. The absence of an appropriate means to assess sequencing error, in a platform independent manner, in the context of metagenomic data, grows more acute with the increasing democratization of high-throughput sequencing technologies (www.technologyreview.com/biomedicine/26850/) and the rapid proliferation of projects that utilize them [21], [22], [23], [24] (in addition, www.1000genomes.org, www.commonfund.nih.gov/hmp, www.earthmicrobiome.org). This includes an increasing trend toward meta-analyses (studies that consider data from multiple sources) to examine collections of samples that can exhibit a diverse technical provenance [25], . Meaningful comparisons of technically diverse sequence data require accurate and platform-independent measures of sequencing error, such that *bona fide* observations can be differentiated from background noise. Current methods, score-based methods in particular, are not well equipped to provide these comparisons.

## Results

### A brief description of Duplicate Read Inferred Sequencing Error Estimation

The limitations of reference-genome and score -based methods inspired the creation of Duplicate Read Inferred Sequencing Error Estimation (DRISEE). DRISEE exploits *artifactually duplicated reads* (ADRs), nearly identical reads that share a completely identical prefix, present with abundances that greatly exceed chance expectations, even when a modest level of biological duplication is taken into account [12], [26]. We exploit the highly improbable abundances of ADRs to distinguish them from other reads (see Methods for details). While 100% identity in the prefix region is used to cluster reads, only the non-prefix bases (those not required to exhibit identity with other reads) are used in the error calculations. No additional requirement for sequence identity/similarity is required of the non-prefix bases. Given their technical origins, it is reasonable to assume that sequence variation within groups of ADRs are more likely to be the product of technical artifact(s) (i.e. sample processing and/or sequencer errors) than a reflection of genuine diversity in the originally sampled population or culture. Based on this premise, DRISEE utilizes multiple alignment (by default, multiple alignments are processed with QIIME [2] integrated Uclust [29] – users will soon be able to choose from a variety of other multiple alignment algorithms) of groups of prefix-identical clusters of ADRs to create internal standards (consensus sequences) to which each individual duplicate read is compared. Sequencing error is determined as a function of the variation that exists within clusters of ADRs. This strategy is platform-independent and can be used to quantify error in metagenomic or genomic samples with respect to error frequency and type. DRISEE identifies duplicate reads using stringent requirements for prefix length and abundance that are extremely unlikely to occur unless the sequences have been artifactually duplicated. In the work presented here, a prefix length of 50 bases and a minimum abundance of 20 reads was used; chance occurrence ≈ 4E-32 (see Methods). It is important to note that this probability is so small as to be deemed effectively impossible in biological sequence data (by way of comparison, the number of atoms in the human body has been estimated at ∼E28 [30]); however, ADRs routinely exhibit abundances that greatly exceed these expectations, making it relatively easy to identify these sequences and simultaneously differentiate them from much lower abundance biological duplication (there are obvious exceptions to this notion, conserved regions in 16s ribosomal genes, repetitions in eukaryotic DNA etc.). Figure 1b provides a visual overview of DRISEE; text S1 (Supplemental Methods) outlines a typical DRISEE workflow in much greater detail.

### DRISEE tables, the preliminary output of DRISEE

**Figure 2:**
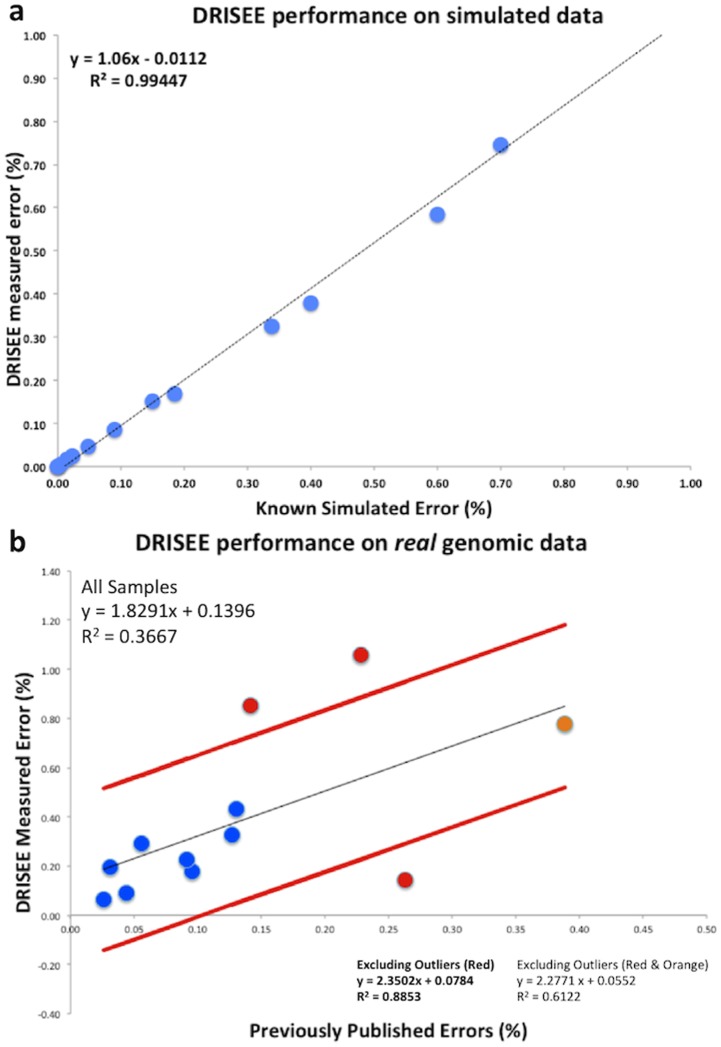
DRISEE performance on simulated and real data. (**a**) Simulated data sets were generated from real whole genome sequences [12], taken from a single sequenced genome, and randomly fragmented into reads that exhibit length distributions consistent with different sequencing technologies (see Methods). Total DRISEE error rates for each sample (Y-axis) are plotted against the known, artificially introduced error rates (X-axis). The equation and R^2^ values represent a linear regression of displayed data. (**b**) DRISEE and a conventional reference-genome-based error method were applied to a set of published genomic data sets [12] (see Methods). Cumulative DRISEE errors (Y axis) are plotted against reference-genome errors determined for the same sample. The equations and R^2^ values represent linear regressions of displayed data. The regression for all samples is plotted as a black line; red lines indicate this regression plus or minus one standard deviation. Red points indicate values further than one standard deviation from the “All Samples” regression. Orange indicates a single point that may disproportionately inflate the observed R^2^. Equations and R^2^ values for the “All Samples” regression are provided as well as for regressions that exclude only the red points or the red and orange points.

**Figure 3:**
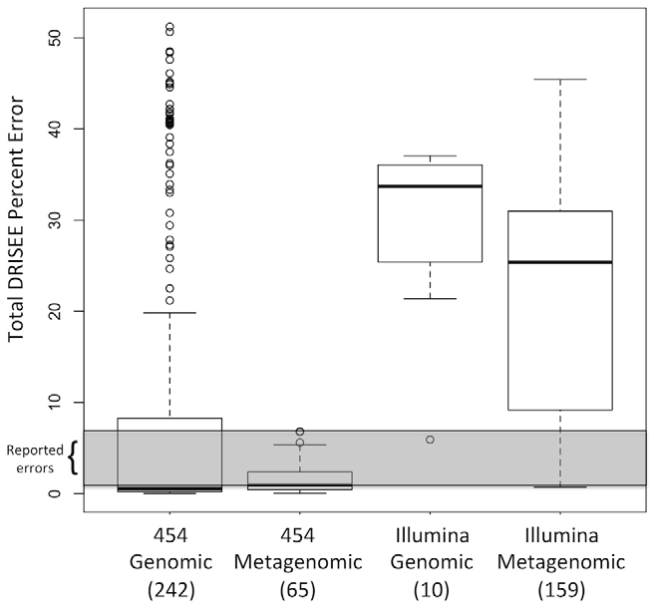
Total DRISEE errors of genomic and metagenomic data produced by 454 and Illumina technologies. A boxplot (conventional five number summary) presents the distribution of averaged total DRISEE errors observed among 476 sequencing samples. The average total DRISEE error is plotted on the Y-axis. X-axis labels indicate the technology (454 or Illumina), type of sample (shotgun genomic or shotgun metagenomic), and in parenthesis, number of samples represented by each individual boxplot. Gray highlight indicates the range of values that have been previously reported for error on 454 and Illumina sequencing platforms (0.25–4%).

**Figure 4:**
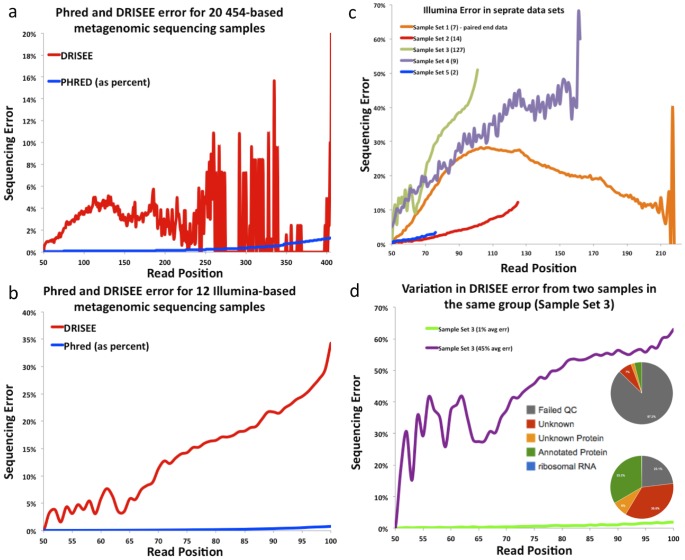
DRISEE error profiles for metagenomic sequencing data sets. Total (% substitutions + % insertions + % deletions) DRISEE error (Y-axis) as a function of read position (X-axis) for all considered reads. (**a**)** and **(**b**)**: Phred vs. DRISEE**: Total DRISEE (red) and average Phred (blue) derived errors (Q values converted to percent error) for (a) 20 metagenomic 454 samples and (b) 12 metagenomic Illumina samples. **(c):** **DRISEE total error of several Illumina-based sample sets**: DRISEE total error profiles are displayed for 5 different Illumina experiments/sample sets. Parentheses indicate the number of samples in each experiment/sample set. **(d):** **DRISEE total error of single samples**: DRISEE total error profiles are displayed for two individual samples. The samples represent the lowest and highest averaged DRISEE total errors (averaged across all read positions), observed in Sample Set 3 (see Figure 4c above). Pie charts indicate a summary of MG-RAST-based annotation of the two samples. The upper pie chart was produced from the data set that corresponds to the purple DRISEE profile (average DRISEE error = 45%). The lower pie chart corresponds to annotation of the data set that produced the green DRISEE profile (average DRISEE error = 1%).

**Figure 5:**
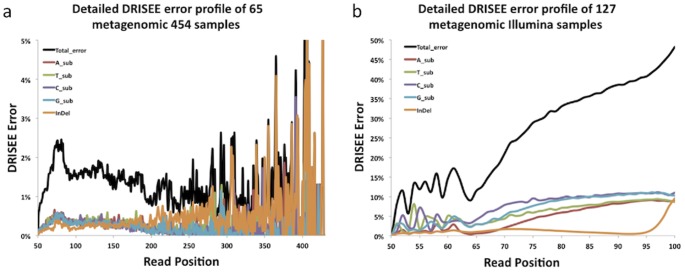
DRISEE calculated Errors, separated by error type, for 454 and Illumina metagenomic samples. DRISEE error profiles are displayed for metagenomic data produced by the 454 (65 samples, (a)) and Illumina (127 samples, (b)) platforms. DRISEE determined errors (Y-axis) are plotted with respect to read position (X-axis). DRISEE errors are displayed as total (black) and type separated (A_sub = A substitutions, T_sub = T substitutions, C_sub = C substitutions, G_sub = G substitutions, and InDel indicates insertions and deletions).

The initial output of a DRISEE analysis is a table, excerpted examples of which are presented as Tables 1 and 2. It indicates the number (Table 1), or percent (Table 2), of sequences (indexed by consensus sequence position) in all considered clusters of ADRs that match or do not match the consensus derived from the ADR cluster to which they belong. DRISEE tables can indicate the match/mismatch counts for a single cluster of prefix-identical reads from a single sequencing sample, for multiple clusters from a single sample (Tables 1 and 2 present one such example), or for multiple clusters collected from a large number of samples that may represent some common trait of interest (e.g. samples produced with the same sequencing technology, that used the same RNA/DNA extraction procedures, that were collected as part of the same sequencing project etc.). This adaptable tabular format represents the simplest incarnation of a DRISEE error profile; it can be analyzed and visualized in a number of ways (numerous examples are presented below – see Figures 2–5) to garner detailed platform-independent information regarding sequencing error in genomic and metagenomic shotgun sequencing data. A more detailed description of the tabular format is included in the legend for Tables 1 and 2.

**Table 1:** Excerpt from a whole sample/run DRISEE error profile table – Raw abundance values.

ID:											
4462612.3											
Summary:											
			A_subst	T_subst	C_subst	G_subst	InDel	Total_err		prefix_length = 50	
			0.1436%	0.0961%	0.1441%	0.0907%	0.0053%	0.4798%			

bp counts:	Match Consensus						Do Not Match Consensus				
Consensus Position Index	A	T	C	G	InDel		A	T	C	G	InDel
1	8646	5849	6119	11508	0		0	0	0	0	0
2	15800	6418	2782	7122	0		0	0	0	0	0
3	6897	15562	2685	6978	0		0	0	0	0	0
4	8820	7475	10374	5453	0		0	0	0	0	0
5	10454	7590	2645	11433	0		0	0	0	0	0
…	…	…	…	…	…		…	…	…	…	…
75	6320	5899	2543	2546	0		5	17	26	1	0
76	5760	5997	2548	2476	0		75	64	67	88	0
77	5912	5884	2431	2579	0		9	3	37	19	0
78	5228	6069	2414	2650	0		38	28	33	53	1
79	5511	5402	2550	2664	0		43	15	27	0	0
…	…	…	…	…	…		…	…	…	…	…

**ID**: Indicates an identification marker for the sample, in this case, an MG-RAST ID.

**Summary**: Indicates the total error as a percent of summed counts for each indicated class of error as well as the average percent error per position for each indicated class of error.

**bp counts**: Each *Consensus Position* numbered row presents the number of matches (*Match Consensus*) and mismatches (*Do Not Match Consensus*) of the indicated variety found across all reads in all considered bins at the indicated consensus position (Consensus Position Index). For example, the value of 8,646 in the *Match Consensus* A column indicates that across all considered bins, 8,646 reads match a consensus A in the first position of their respective bin consensus.

**Table 2:** Excerpt from a whole sample/run DRISEE error profile table – Percent scaled abundance values.

ID:4462612.3											
Summary:											
			A_subst	T_subst	C_subst	G_subst	InDel	Total_avg_err		prefix_length = 50	
			0.203%	0.151%	0.226%	0.191%	0.001%	0.772%			

bp counts:	Match Consensus						Do Not Match Consensus				
Consensus Position Index	A	T	C	G	InDel		A	T	C	G	InDel
1	26.916%	18.209%	19.049%	35.826%	0.000%		0.0000%	0.0000%	0.0000%	0.0000%	0.0000%
2	49.187%	19.980%	8.661%	22.172%	0.000%		0.0000%	0.0000%	0.0000%	0.0000%	0.0000%
3	21.471%	48.447%	8.359%	21.723%	0.000%		0.0000%	0.0000%	0.0000%	0.0000%	0.0000%
4	27.458%	23.271%	32.296%	16.976%	0.000%		0.0000%	0.0000%	0.0000%	0.0000%	0.0000%
5	32.545%	23.629%	8.234%	35.592%	0.000%		0.0000%	0.0000%	0.0000%	0.0000%	0.0000%
…	…	…	…	…	…		…	…	…	…	…
75	36.412%	33.986%	14.651%	14.668%	0.000%		0.0288%	0.0979%	0.1498%	0.0058%	0.0000%
76	33.734%	35.122%	14.922%	14.501%	0.000%		0.4392%	0.3748%	0.3924%	0.5154%	0.0000%
77	35.036%	34.870%	14.407%	15.284%	0.000%		0.0533%	0.0178%	0.2193%	0.1126%	0.0000%
78	31.658%	36.751%	14.618%	16.047%	0.000%		0.2301%	0.1696%	0.1998%	0.3209%	0.0061%
79	33.993%	33.321%	15.729%	16.432%	0.000%		0.2652%	0.0925%	0.1665%	0.0000%	0.0000%
…	…	…	…	…	…		…	…	…	…	…

**ID**: Indicates an identification marker for the sample, in this case, an MG-RAST ID.

**Summary**: Indicates the total error as a percent of summed counts for each indicated class of error as well as the average percent error per position for each indicated class of error.

**bp counts**: Each *Consensus Position* numbered row presents the percent of matches (*Match Consensus*) and mismatches (*Do Not Match Consensus*) of the indicated variety found across all reads in all considered bins at the indicated consensus position (Consensus Position Index). For example, the value of 26.916% in the *Match Consensus* A column indicates that across all considered bins, 26.916% reads match a consensus A in the first position of their respective bin consensus.

### Validation of DRISEE with simulated and real sequencing data; Comparison of DRISEE to reference-genome-based estimation of sequencing error

Initial validations of DRISEE with simulated data showed nearly perfect correlations between known and DRISEE-based error estimates (Figure 2a, R^2^ = 0.99). Additional validations with real genomic sequencing data exhibit good correlation with error estimates produced by conventional reference-genome-based analyses [12] of the same samples (Figure 2b, R^2^ = 0.89, excluding outliers).

### DRISEE reveals unexpected levels of error in genomic and metagenomic data from two widely utilized high-throughput sequencing technologies

In further trials, DRISEE was applied to genomic and metagenomic shotgun data produced by two widely utilized sequencing technologies, 454 and Illumina (n = 242 genomic 454, n = 65 metagenomic 454, n = 10 genomic Illumina, and n = 159 metagenomic Illumina samples), 476 samples in all. Less than half of the individual samples (*n* = 169) exhibit DRISEE-based errors consistent with the reported range of second-generation sequencing errors (0.25–4%) [4], [11], [12], [13], [19], [31]. The majority of samples (*n* = 307) exhibit DRISEE-based errors that fall outside the range of reported sequencing errors (error<0.25%, *n* = 73; error>4%, *n* = 234; avg ± stdev = 12.63±15.12) (Figure 3). The Supplemental Methods (Text S1) include a description as to how data sets were selected.

### DRISEE detects error levels much higher than those produced by a conventional score-based approach; Comparison of DRISEE to Phred-based estimation of sequencing error

To compare DRISEE derived errors with those determined with a more conventional score-based approach, we obtained FASTQ data (i.e. Phred scores) via SRA (http://www.ncbi.nlm.nih.gov/sra) for subsets of DRISEE-analyzed samples: 20 of the 65 metagenomic 454 samples and 12 of the 159 Illumina metagenomic samples. Per base DRISEE and Phred [32]-based errors for these samples were calculated and compared (see Methods). In 454 and Illumina-based metagenomic sequencing data, DRISEE profiles reveal error levels much higher than those reported by archived Phred values (Figure 4a & b). It is also intriguing to note that, whereas Phred values exhibit nearly indistinguishable trends between the 454 and Illumina data, DRISEE error profiles differ markedly for each technology (Figure 4a & b).

### DRISEE reveals drastic differences in sequencing error among experiments and even between individual samples from the same experiment

After observing differences in error profiles between 454 and Illumina technologies, we explored the possibility that DRISEE could be used to observe differences in sequencing error produced by a single sequencing platform (Illumina). Sequencing samples from five projects (i.e. groups of samples that were produced together in a single experimental framework) were explored by comparing the total DRISEE error profile for each (Figure 4c). While two projects exhibited similar error profiles (Sample Sets 2 and 5), most were unique. The ability of DRISEE to resolve unique error profiles was tested further by exploring two individual samples taken from the same project/experiment (Sample Set 3), those that exhibited the highest and lowest average DRISEE errors. Although the two samples were produced on the same sequencing platform as part of the same experimental project, the individual error profiles are drastically different (Figure 4d). The two samples underwent annotation via MG-RAST, a summary of the annotation results for each sample appears, as a pie-chart, imbedded in the plot of the DRISEE profiles.

### DRISEE provides detailed data regarding error type

We also used DRISEE to provide data regarding error type. Figure 5 presents all error types together (total error) as well as a breakdown of each error type (A,T,C, and G substitutions and insertion/deletion errors) observed across metagenomic 454 (65 samples) and Illumina (159 samples) data. The results are consistent with previous observations in genomic shotgun sequencing: Illumina data are dominated by substitution-based errors [14], whereas 454 data exhibit a majority of insertion/deletion errors [13] (Figures 5a and 5b). No other method provides estimates with respect to error type in metagenomic shotgun data.

## Discussion

DRISEE provides a more complete estimate of sequencing error than is possible with score-based methods, one that accounts for error introduced at any/all procedural steps in a sequencing protocol – all steps that have the potential to introduce errors (i.e. deviation from the original biological template sequences) – from collection of a biological sample to extraction of DNA/RNA, intermediary processing of the extracted material and, finally, sequencing itself (see Figure 1a). Error introduced by processes outside of the actual act of sequencing are ignored by score-based methods, thus it is not surprising that DRISEE derived errors are generally larger than Q/Phred scores, as they account for errors introduced over a much broader scope of experimental procedures, from sample collection, to a wide variety and number of possible intermediary processes, to sequencing itself. An example may help to illustrate the critical importance of this consideration:

Amplification is commonly utilized to generate sufficient quantities of material for sequencing from an initial RNA/DNA sample. Here we refer specifically to amplification performed outside of the sequencer/sequencing protocol. Various methods exist – classically variants of the polymerase chain reaction were used, more recent incarnations have adopted isothermic techniques – all depend on high fidelity enzymes (e.g. Taq or **Φ**29 DNA polymerase), and are experimental processes, prone to experimental error. Even with high fidelity enzymes, amplification products will contain errors (i.e. deviations from the original biological template). Successive amplification(s) propagate previous errors and introduce new ones, leading to populations of reads that increasingly diverge from their original biological templates. Amplification products are frequently used as the starting material for a sequencing run, thus the starting material may contain large numbers of unique reads that do not exist in the original biological sample. Score-based methods have no means to distinguish these unique and non-biological reads from the original templates. Scores do provide useful information, the fidelity with which sequencer base calls are made, but these estimates possess no information with respect to the origin of the sequenced read: is the sequence genuine/biological or an error containing artifact of imperfect amplification? Through the careful examination of prefix-identical reads, DRISEE is able address this question; in the context of shotgun metagenomic data, no other method can.

We assert that reference-genome-based error determination methods provide the most complete and accurate measure of sequencing error. This is due to the fact that (1) such methods consider the entire scope of procedures that accompany a typical sequencing experiment and (2) they compare raw sequence data to an absolute standard, a reference genome. Score-based metrics (e.g. Q or Phred) only consider error introduced by the actual act of sequencing (ignoring error introduced by any processes that precede actual sequencing – e.g. DNA/RNA extraction, sample amplification and purification, etc.) and are the product of proprietary black-box software products that can vary considerably among different sequencing technologies. Unfortunately, reference-genome-based methods cannot be applied to metagenomic data (reference metagenomes do not exist, and are unlikely to anytime in the near future). DRISEE can be thought of as a reference-genome-like method, the key difference is that the reference sequences are derived internally from the pool of artifactual duplicate reads, and not from an external reference genome. The similarity between reference-genome and DRISEE derived errors for the same genomic sequencing samples (Figure 2b) is not surprising; both methods rely on comparisons to reference standards. Unfortunately, reference-genome-based methods cannot be applied to metagenomic data (the appropriate reference standards do not exist).

Reference-genome-based methods possess another potential fault, the utilization of preliminary identity/similarity filters that may lead to artifactual deflation of error estimates. In particular, conventional reference-genome-based methods employ a preliminary similarity search to align sequenced reads to the most similar portion of the selected reference genome. Reads that fail to align to the reference genome with the selected initial level of stringency (criteria are generally lenient, e.g. 90% identity for the full length of the read [12]) are discarded from subsequent analysis. In this way, the most error prone reads, those that do not align well to the reference genome, even with lenient criteria, and would contribute significantly to calculated error, are not considered. DRISEE takes a very different approach. Reads are binned based on 100% identity in their prefix region, but no identity/similarity requirement is made of the non-prefix bases. Criteria for prefix length and abundance provide conditions so improbable as to preclude any possibility other than technical duplication. Technical duplicates should be identical to each other, not just in their prefix region, but through the length of the entire read, except for differences introduced by error. While 100% identity in the prefix region is used to cluster reads, only the non-prefix bases (those not required to exhibit identity/similarity with other reads) are used in the error calculations. As no additional requirement for sequence identity/similarity is required of the non-prefix bases, DRISEE can provide estimates of error that are less constrained by filters placed in conventional reference-genome based methods. As an example, consider a 100 bp read. Under the reference-genome-based method utilized by Niu et al. (see Figure 2b), 90 bp would be required to perfectly align with a reference genome before error analyses are conducted; thus, the maximum detectable deviation from the reference standard is 10% (i.e. a maximum of 10% error can be detected). Alternatively, DRISEE would cluster the read into a bin of reads with the same 50 bp prefix and would subsequently ignore this prefix to produce an estimate of error solely on the non-prefix bases (those not required to exhibit identity/similarity with other reads in their respective bin). This allows DRISEE to consider errors that span a much broader range (errors in excess of 50% have been observed – see Figure 3).

Given that DRISEE considers the complete scope of procedures implemented in a given sequencing experiment, and score-based methods only provide information with respect to the actual act of sequencing, it is not surprising that DRISEE produces error estimates that are generally higher (Figures 3, 4a, & 4b). The uniqueness of DRISEE error profiles was unexpected. Distinct error profiles are observed for each of two sequencing technologies, 454 (Figure 4a) and Illumina (Figure 4b); each exhibits a clearly unique error profile, whereas Q-value derived error profiles for the very same samples are indistinguishable from each other. Furthermore, unique profiles were observed when samples processed with the same sequencing technology (Illumina) were grouped by experiment, suggesting the presence of platform-independent technological or lab-dependent errors (Figure 4c). Even finer distinctions are observable among the error profiles for single samples taken from the same experiment (Figure 4d).

DRISEE provides a means to assess the relative quality of sequencing between technologies (Figure 4a and b), experiments performed on the same platform (Figure 4c), and even between individual samples taken from the same experiment (Figure 4d). The ability of DRISEE to provide a preliminary estimate of sample quality, and indications as to the suitability of a sample for subsequent analyses, is clearly demonstrated in Figure 4d. Two samples from the same experiment exhibit vastly different DRISEE error levels (1 vs. 45% average error). These values are reflected in the MG-RAST-based annotations of the samples. Nearly 90% of the reads from the high error sample fail MG-RAST quality control procedures; just 4% of the reads are successfully annotated as known proteins. The higher quality data set loses a much smaller portion of its reads to quality control (23%) and has eight times as many reads annotated as known proteins (33%).

In the current age of compute-constrained bioinformatics, the identification and correction/removal of low quality sequence data, from relatively mild procedures like read trimming – DRISEE informed read trimming is currently under development – to more drastic action, including the elimination of entire sequencing samples, is an acute and steadily growing necessity. DRISEE can provide researchers with the ability to identify low quality sequence data before time-consuming and potentially costly analyses are performed. DRISEE also provides researchers with a platform-independent means to assess error among samples, after they have undergone analyses, allowing a quantitative assessment as to the fidelity of analysis-derived inferences. As an example, annotations related to high error samples like that presented in Figure 4d (purple DRISEE profile) should be treated with a great deal more skepticism than those derived from a higher quality data set (e.g. 4d, green DRISEE profile). This is especially true when considering samples with subtle differences that may easily be obscured by high levels of sequencing error.

Arguably, DRISEE has some limitations. At present, it is not applicable to eukaryotic data, sequences with low complexity, and/or known sequences that may exhibit an unusually high level of biological repetition, particularly amplicon ribosomal RNA data. These types of data are likely to meet DRISEE requirements for prefix length and abundance, but represent real biological variation that could be misinterpreted by DRISEE as sequencing error. Moreover, DRISEE operates on artifactually duplicated reads—an approach that works well with current platforms such as 454 and Illumina but may require procedural modifications (such as the intentional inclusion of highly abundant sequence standards) if future developments eliminate ADRs.

In summary, DRISEE provides accurate assessments of sequencing error of metagenomic (Figures 3–5) and genomic (Figure 2) data, accounting for error type as well as frequency (Figure 5). DRISEE error profiles can be used to explore correlations between sequencing error and metadata (e.g. Figure 4a & b suggests the presence of platform dependent trends in DRISEE calculated errors; Figure 4d demonstrates a correlation between DRISEE calculated error and the percent of reads that MG-RAST is able to successfully characterize), allowing investigators to differentiate experimentally meaningful trends from artifacts introduced by previously uncharacterized sequencing error. Traditional score- and reference-genome- based methods do not allow for such observations with respect to shotgun metagenomic data. DRISEE also offers the advantage that it requires no data other than an input FASTA or FASTQ file. Moreover, DRISEE considers error independent of sequencing platform, without prior knowledge. These characteristics make DRISEE a promising method—particularly with respect to the enormous quantities of shotgun-based metagenomic data that are anticipated in the near future.

DRISEE will soon be available to analyze sequencing samples in MG-RAST. We also provide MG-RAST independent code to allow users to perform DRISEE analyses without MG-RAST: https://github.com/MG-RAST/DRISEE.

## Methods

### Overview

Duplicate Read Inferred Sequencing Error Estimation (DRISEE) can be applied to sequence data produced from any sequencing technology. It provides an error profile (Tables 1 and 2 provide an excerpted example) that can be used to explore the sequencing error, as well as biases in error, that are present in a single sequencing run or any group of sequencing runs. The latter capability enables the user to produce error profiles specific to a particular sequencing technology, sample preparation procedure, or sequencing facility—in short, to any quantified variable (i.e., metadata) related to one or more sequencing samples.

DRISEE exhibits several desirable characteristics that are not found in the most widely utilized methods to quantify sequencing error: *reference-genome-based methods* that rely on comparison to standard sequences (generally a published sequenced genome): and quality *score-based* methods that rely on sophisticated, platform-dependent models of error to derive base calls with affiliated confidence estimates (Q or Phred scores) for each sequenced base. DRISEE can be applied to metagenomic or genomic data produced with any sequencing technology and requires no prior knowledge (i.e., reference genomes or platform–dependent error models).

DRISEE relies on the occurrence of artifactually duplicated reads—nearly identical sequences that exhibit abundances that greatly exceed expectations of chance, even when a modest amount of possible biological duplication is taken into account. Illumina and 454 platforms exhibit a well documented [12], [26], but poorly understood, propensity to produce large numbers of ADRs. DRISEE utilizes this artifact as a means to create internal sequence standards that can be used to assess error within a single sample, or across multiple samples. We identify ADRs as those reads that exhibit an identical prefix (prefix = the first *l* bases of a read) at some threshold abundance (*n*) that exceeds chance expectations, even those that take biological duplication into account. The precise values of *l* (prefix length) and *n* (prefix abundance) can be varied to accommodate the scale of any sequencing technology. In the work presented here, bins (groups) of duplicate reads were used to calculate error values if they exhibited an identical prefix length (*l*) of 50 bases with an abundance (n) of 20 or more reads. These requirements are arbitrary, but were selected on sound statistical and biological assumptions. Chief among these is the extreme improbability that such an occurrence (20 reads, each with identical 50 bp prefixes) could occur by chance, (i.e. without technical duplication via WGA or PCR etc. These criteria are stringent enough to justify assumptions of biological and statistical uniqueness; indeed, such an occurrence is extremely unlikely by chance:
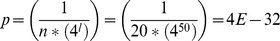
where *p* is the probability that a prefix of length *l* (50 bp) will be observed *n* (20) times; 4 represents the number of possible bases (A, T, C, and G). Even in data that are Illumina scale (on the order of 1 million reads per run), a chance observation of 20 reads that exhibit the same 50 bp prefix is highly improbable (chance≈1E06×4E-32 = 4E-26); however, ADRs frequently exceed these limits, making them easy to detect, and providing an ideal population to probe for sequencing error – a population of reads that should be completely identical (i.e. identical beyond their 50 bp prefix) except for errors introduced by sequencing procedures. The default values for nucleotide length and number of reads required for a bin of ADRs to undergo DRISEE analysis are arbitrary; however, they possesses a key feature, improbability far beyond that expected by chance, even if biological repetition was present, and even when data are Illumina scale (1E06 reads). Less stringent criteria (prefix length 20 bp, prefix abundance 20; *p* = 5E-14) were applied to data generated by 454 technologies, yielding extremely similar estimations of error (data not shown). Much more stringent criteria were selected for this study such that the method could be applied to 454 and Illumina data without concern for the difference in scale in the outputs of the two technologies (454≈1E05, Illumina≈1E06 reads per run).

DRISEE exhibits a universality that other methods lack, but only if the data under consideration meet the following criteria: (1) Data must be in FASTA or FASTQ format. (2) There must be enough ADRs to safely infer that they are the product of artifact and not of real biological variation. (3) Input sequence data should not be culled, trimmed, or modified in any way by sequencer processing software: note that while DRISEE utilizes ADRs in its calculations, it does not cull these sequences from processed datasets (4) Data under consideration should be the product of random (i.e. shotgun) sequencing. (5) At this time, amplicon data—specifically, directed sequencing of amplicon ribosomal RNA data, are not suitable for DRISEE analysis; ribosomal amplicon reads start with highly conserved regions (primer target sites) followed by regions that exhibit a large degree of real biological variation (the hypervariable regions) that DRISEE could misinterpret as error.

### Data access

Unless otherwise indicated, data sets examined in this study were obtained via SRA or MG-RAST. Table S1 (Supplemental Table 1) contains a complete list of sequence data used in the accompanying manuscript. Datasets are referenced by their SRA (http://www.ncbi.nlm.nih.gov/sra), MG-RAST (http://metagenomics.anl.gov/), or both identifiers/accession numbers.

An MG-RAST independent version of DRISEE code, with detailed documentation, including installation and running instructions as well as runtime related statistics, can be downloaded from https://github.com/MG-RAST/DRISEE.

See **Text S1 (Supplemental Methods)** and **Figure 1b** for a detailed workflow-based description of DRISEE.

### Table 1 and 2 overview

DRISEE analysis tables take the same form if they exhibit the counts derived from a single bin of artificially duplicated reads, multiple bins from the same sample, or much larger collections of bins spanning multiple samples. The excerpted tables displayed here represent the raw and percent scaled DRISEE error profile for all considered prefix-identical bins in a single metagenomic sequence sample (MG-RAST ID 4462612.3). The DRISEE table is presented as raw counts per base pair position (Table 1) or percent error per position (Table 2). Tables 1 and 2 contain three sections (ID, Summary, and bp counts), described in the legends below.

## Supporting Information

Table S1 Supplemental Table 1 contains a complete list of MG-RAST and/or SRA accession numbers for all data used in this study.
(XLS)

Text S1 Supplemental Methods. Contains an extended workflow description of a typical DRISEE analysis and some additional detailed descriptions of methods briefly referred to in the main text.
(DOC)
