# Supplementary material for: A Platform-Independent Method for Detecting Errors in Metagenomic Sequencing Data: DRISEE
Source: PLoS Comput Biol. 2012 Jun 7;8(6):e1002541. doi: 10.1371/journal.pcbi.1002541 (PMC3369934; doi:10.1371/journal.pcbi.1002541)
Supplement: Text S1 — Supplemental Methods. Contains an extended workflow description of a typical DRISEE analysis and some additional detailed descriptions of methods briefly referred to in the main text. (DOC) [file pcbi.1002541.s002.doc]

**Typical DRISEE workflow (see Figure 1b):**

DRISEE was designed primarily as a means to assess metagenomic sequencing error in samples submitted to MG-RAST. DRISEE will soon be available via MG-RAST-based analyses of shotgun metagenomic data. We also provide MG-RAST independent code that will allow users to perform DRISEE analyses on their own. Below we outline the steps in a typical DRISEE workflow. All analyses presented in the accompanying manuscript utilized the default values and arguments mentioned below.

**1. Sequence data in FASTA format**

In an effort to keep DRISEE as platform-independent as possible, it considers data in nearly ubiquitous FASTA format. Tools are described elsewhere for conversion to FASTA (http://maq.sourceforge.net/fq_all2std.pl). DRISEE specifically avoids use of Phred scores or any other platform-dependent error metric as a means to sort, cull, trim, or assess reads. Typical input includes all sequenced reads produced in a single sequencing run or sample.

**2. Check for random sequencing**

DRISEE was designed to consider sequencing data generated by random (shotgun) procedures. While it can be used to explore variation in amplicon-based data, such variation cannot be equated with sequencing error. Amplicon-based data are identified either through available metadata or the application of a tool that identifies amplicon and/or randomly sequenced samples based on an analysis of their prefix entropy (i.e. the Shannon entropy of the distributions of prefixes of successive lengths). Samples that exhibit nonrandom sequence patterns in their prefix, consistent with expectations of amplicon samples, are excluded from consideration.

**3. Screen reads for length and the presence of ambiguous base calls**

Sequence data undergo a mild two-step filtering procedure to remove two types of reads:

A. Reads that exhibit uncharacteristic lengths (the default setting is to remove those that exhibit lengths farther than two standard deviations from the mean).

B. Reads with ambiguous base calls (the default setting is to remove reads that contain any ambiguous base calls).

**4. Bin reads based on the presence of identical prefixes**

A list of unique prefixes in the screened FASTA file is generated. All remaining reads are then grouped (or binned) according to their prefix. Any prefix length can be used; the current default is 50 bases.

**5. Screen bins by abundance**

Groups (or bins) of prefix-identical reads are sorted with respect to read abundance. Bins with a minimum number of reads are processed further. Any minimum can be used; the current default is 20 reads. DRISEE can consider all such bins in a sample, or can consider any arbitrary number of such bins. DRISEE analyses presented in the accompanying manuscript considered no more than 1,000 randomly selected bins for each sample. DRISEE can use all reads in a given bin, or any arbitrary number of reads that is equal to or larger than the required minimum number of reads. DRISEE analyses presented in the accompanying manuscript considered all reads for bins that contained between 20 and 1,000 reads. A random selection of 1,000 reads was considered for any bin that had more than 1,000 reads. Results derived from complete DRISEE analyses (consideration of all reads in all bins that met DRISEE requirements) and analyses that used a maximum bin limit of 1,000 with a read limit of 1,000 exhibited little appreciable difference (data not shown). A variety of alternative selection and/or bootstrap procedures are possible with DRISEE analyses—these are not discussed here.

**6. Screen for eukaryotic or other suspect content**

Bins can be screened for eukaryotic content, sequences with low complexity, and/or known sequences that may exhibit an unusually high level of biological repetition (16s rRNA-based, sequences with low complexity, eukaryotic sequences etc.). Bins that contain such sequences should be excluded from further consideration.

**7. End-trim reads**

Reads within a bin can be trimmed to a uniform length; the default operation is to trim reads to the length of the shortest read in their respective bin. Trimming can be performed by using other criteria or can be avoided altogether.

**8. Construct consensus sequences via multiple alignment**

All reads for a given bin undergo multiple alignment. As of the most current version, DRISEE utilizes QIIME integrated Uclust , with additional custom scripts to perform multiple alignments. Once computed, multiple alignments are used to generate a consensus (i.e. Steiner) sequence. In the first iteration, a seed sequence is randomly chosen from all reads in the bin. In the second and all subsequent iterations, the consensus sequence generated from the previous iteration is used as the seed. This process is iterated until it achieves convergence (defined as three consecutive iterations with no change in cluster identity) or once a pre-determined iteration limit has been achieved (by default 10; in practice, bins rarely require more than the first two to three iterations to achieve convergence).

**9. Compare bin reads with the bin consensus sequence**

Each individual read in a bin is compared, base-by-base, with the final consensus sequence for that bin. Only the non-prefix portion of the reads is considered (bases 51 and onwards in the work presented in the accompanying manuscript). At each position (with respect to the consensus sequence) a read is scored as one of six matches (A, T, C, G, insertion, or deletion) or one of six mismatches (A substitution, T substitution, C substitution, G substitution, insertion, or deletion). Insertions and deletions that span more than one base are scored as multiple mismatches (e.g. a deletion of three bases is scored as three mismatches).

**10. Construct bin-level DRISEE profiles**

Deviations and matches for all reads in a bin are tallied with respect to position in the consensus sequence. The consensus position indexed table of matches and mismatches for the bin represent its bin-level DRISEE profile.

**11. Construct run- or sample-level DRISEE profiles**

DRISEE profiles for all considered bins in a given run can be combined (summed) to produce a DRISEE error profile for the entire run or sample (see Tables 1 and 2 for examples).

**12. Sample Group level DRISEE profile construction**

DRISEE profiles for all bins in a group of runs can be combined (summed) to produce a DRISEE error profile for any group of runs/samples.

DRISEE profiles and the data they contain can be visualized directly, or processed further to generate detailed analyses of the information they contain, like those presented in this manuscript (see Figures 2-5).

**Construction and Analysis of Simulated Data**

Datasets with known rates of error were generated with Metasim. Each artificial data set contained 100,000 sequences (average length of 252bp) randomly selected from the first 2,500 bases of the *E. coli* K-12 genome (Refseq accession number NC_000913) and randomly corrupted with errors of known type and frequency. Data sets represented a variety of substitution (0 to 2.3%) and insertion and deletion (0 to1.5% each) rates. Data sets contained substitution only, insertion and deletion only, or a combination of error types. Simulated data sets were processed with DRISEE as if they were real data sets. DRISEE detected errors were compared with known simulated rates, with respect to frequency and type (see Figure 2a).

**Comparative Analyses of Real Genomic Sequencing Data**

Real genomic sequencing data were acquired from a previously reported study that used reference-genome-based methods to explore sequencing error in several single genome sequencing samples. DRISEE was applied to data sets only if they met the criteria used with all other data considered in the accompanying manuscript: one or more bin(s) of reads that exhibit 20 or more reads with an identical 50-base prefix. A total of 12 samples obtained from SRA (http://www.ncbi.nlm.nih.gov/sra) met these criteria: SRR013470, SRR001574, SRR007446, SRR013433, SRR013137, SRR000266, SRR006411, SRR018125, SRR017783, SRR013477, SRR013431, SRR013382. These samples were analyzed with the a reference-genome-based method as previously described and with DRISEE. Errors derived from the originally reported reference-genome-based method and DRISEE were compared (see Figure 2b).

**References**

1. Shannon CE (1997) The mathematical theory of communication. 1963. MD computing : computers in medical practice 14: 306-317.

2. Huse SM, Huber JA, Morrison HG, Sogin ML, Welch DM (2007) Accuracy and quality of massively parallel DNA pyrosequencing. Genome Biol 8: R143.

3. Kunin V, Engelbrektson A, Ochman H, Hugenholtz P (2010) Wrinkles in the rare biosphere: pyrosequencing errors can lead to artificial inflation of diversity estimates. Environmental microbiology 12: 118-123.

4. Caporaso JG, Kuczynski J, Stombaugh J, Bittinger K, Bushman FD, et al. (2010) QIIME allows analysis of high-throughput community sequencing data. Nature methods 7: 335-336.

5. Edgar RC (2010) Search and clustering orders of magnitude faster than BLAST. Bioinformatics 26: 2460-2461.

6. Richter DC, Ott F, Auch AF, Schmid R, Huson DH (2008) MetaSim: a sequencing simulator for genomics and metagenomics. PloS one 3: e3373.

7. Niu B, Fu L, Sun S, Li W (2010) Artificial and natural duplicates in pyrosequencing reads of metagenomic data. BMC Bioinformatics 11: 187.
